# Reverse transcriptase inhibition potentiates target therapy in BRAF-mutant melanomas: effects on cell proliferation, apoptosis, DNA-damage, ROS induction and mitochondrial membrane depolarization

**DOI:** 10.1186/s12964-020-00633-7

**Published:** 2020-09-15

**Authors:** Luigi Fattore, Debora Malpicci, Ciro Milite, Sabrina Castellano, Gianluca Sbardella, Gerardo Botti, Paolo A. Ascierto, Rita Mancini, Gennaro Ciliberto

**Affiliations:** 1 Istituto Nazionale Tumori IRCCS, “Fondazione G. Pascale”, Naples, Italy; 2 Present Address: SAFU Laboratory, Department of Research, Advanced Diagnostics and Technological Innovation, Translational Research Area, IRCCS Regina Elena National Cancer Institute, 00144 Rome, Italy; 3 Department of Experimental and Clinical Medicine, University “Magna Graecia” of Catanzaro, Catanzaro, Italy; 4 Department of Pharmacy, Epigenetic Med Chem Lab, University of Salerno, Fisciano, SA Italy; 5 Department of Molecular and Clinical Medicine, University of Roma “Sapienza”, Rome, Italy; 6 IRCCS, Istituto Nazionale Tumori “Regina Elena”, Via Elio Chianesi 53, 00144 Rome, Italy

**Keywords:** Melanoma, Reverse transcriptase inhibitors, Target therapy, Drug resistance, Mitochondrial membrane depolarization, DSBs

## Abstract

**Abstract:**

Target therapies based on BRAF and MEK inhibitors (MAPKi) have changed the therapeutic landscape for metastatic melanoma patients bearing mutations in the BRAF kinase. However, the emergence of drug resistance imposes the necessity to conceive novel therapeutic strategies capable to achieve a more durable disease control. In the last years, retrotransposons laying in human genome have been shown to undergo activation during tumorigenesis, where they contribute to genomic instability. Their activation can be efficiently controlled with reverse transcriptase inhibitors (RTIs) frequently used in the treatment of AIDS. These drugs have demonstrated anti-proliferative effects in several cancer models, including also metastatic melanoma. However, to our knowledge no previous study investigated the capability of RTIs to mitigate drug resistance to target therapy in BRAF-mutant melanomas. In this short report we show that the non-nucleoside RTI, SPV122 in combination with MAPKi strongly inhibits BRAF-mutant melanoma cell growth, induces apoptosis, and delays the emergence of resistance to target therapy in vitro. Mechanistically, this combination strongly induces DNA double-strand breaks, mitochondrial membrane depolarization and increased ROS levels. Our results shed further light on the molecular activity of RTI in melanoma and pave the way to their use as a novel therapeutic option to improve the efficacy of target therapy.

Video Abstract

**Graphical abstract:**

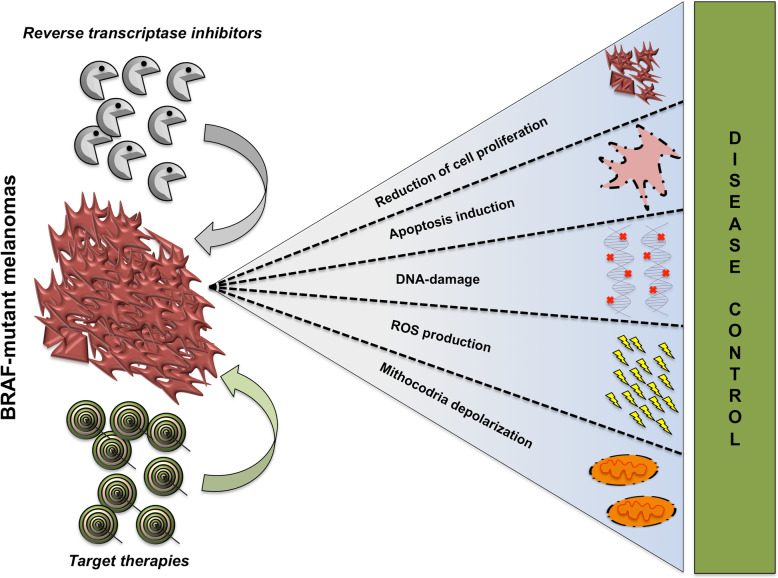

## Background

Combination therapy with BRAF and MEK inhibitors (MAPKi) has become standard of care for melanoma patients (approximately 50%) harboring BRAF-V600 mutations [1, 2]. This therapeutic approach results in rapid and durable objective responses in the majority of patients and in prolonged overall survival. A big issue, however, still remains the emergence of drug resistance [3–6]. From here, the need to identify novel and more efficient combinatorial approaches capable to control the development of drug resistant clones and to avoid disease relapse [7]. Towards this goal, our group has worked in the last years to the identification of non-mutational mechanisms involved in the acquisition of drug resistance. In this context we reported that monoclonal antibodies targeting ErbB3, a member of EGFR family, are able to inhibit activation of this receptor as a mechanism of escaping from MAPKi in melanoma and to delay the emergence of drug resistance in vitro and in vivo [8]. In addition, we have also demonstrated that microRNAs are key players of resistance to target therapy in melanoma and that their targeting is able to restore drug sensitivity [9–11].

A challenging field of cancer research is the possibility to take advantage of reverse transcriptase (RT) inhibitors [12], normally used against retroviruses like HIV-1 for the therapy of AIDS [13]. The rationale for this approach lays on the existence in our genome of retrotransposons, i.e. genetic sequences able to copy themselves into an RNA intermediate and to insert elsewhere in host DNA [14]. The control of the stability of these genomic “parasites” is pivotal for the cellular physiology of the host. There are two classes of retrotransposons in mammals: 1) LTR and 2) non-LTR [14]. Human endogenous retroviruses (HERVs) are members of the first class (LTR) and are remnants of retroviral germline infections and due to a strict epigenetic regulation they are barely expressed in adult healthy tissues [15]. The molecular mechanisms encompassing HERV insertions in genome resemble those used by exogenous retroviruses [16]. Many studies have linked increased HERV expression to tumors, like breast cancer, melanoma and kidney cancer. These results may indicate their oncogenic role, despite causative involvement of HERVs has not still clearly demonstrated [15]. As to non-LTR retrotransposons, they are mostly represented by Long Interspersed Element-1 s (LINE-1 s), which are the only active autonomous retrotransposons in humans. Again, their involvement as passengers or drivers of human cancers is still debated [17]. A proof of concept of their key role in cancer is the evidence that LINE-1 promoters are frequently silenced by methylation in normal cells and then activated by genome-wide hypomethylation during tumorigenesis; an event underpinning high retrotransposon activity and genome instability [18]. In a recent work, retrotransposon activity has been investigated through whole genome sequencing (WGS) in colorectal cancer. This work led to the identification of variable RT insertions in at least 15 known cancer genes. Among them, LINE-1 insertions were observed in the oncosuppressor Adenomatous Polyposis Coli (APC) leading to its inactivation, which suggests a direct contribution to tumor-initiation [19]. Of note, similar findings have been previously observed in colorectal cancer [20]. Finally, an additional class of reverse transcriptase in eukaryotic cells is represented by telomerases (TERT), which are involved in the maintenance of chromosome ends [21]. In most adult human tissues, telomerase activity is low or undetectable. Differently, their up-regulation is a critical event in over 90% of cancers but the molecular mechanisms underpinning TERT activation are not completely understood [22].

Both nucleoside RT inhibitors (NRTIs) and non-nucleoside RT inhibitors (NNRTIs) have demonstrated to be effective anticancer agents in several carcinoma cell lines [23–26]. Inspired by the combined structure-activity relationships of F2-DABO class of NNRTIs and of the related ADATs [27, 28], a series of novel pyrimidinone derivatives were designed and screened for their antiproliferative effects in cancer cell lines. Among them, compound SPV122 demonstrated anti-proliferative effects on melanoma and on prostate cancer cells [29, 30]. Based on these findings we decided to investigate the capability of SPV122 to potentiate target therapy for BRAF-mutant melanomas and to delay the emergence of drug resistance. Furthermore, we have also deepened our knowledge on the molecular mechanisms responsible for the anti-proliferative and apoptotic effects of RTi + MAPKi for the management of metastatic melanoma.

## Materials and methods

### Cell culture, treatments, and reagents

Human melanoma cell lines M14, A375 and WM115 were obtained as previously described [9, 12] and cultured in RPMI supplemented with 10% FBS. For combination assays, BRAFi has been used starting from the highest dose of 5 μM and then diluted 1:2 for 10 times; SPV122 was used at fixed dose of 1.25 μM. All the other experiments have been performed with the following doses: 150 nM for BRAFi, 75 nM for MEKi and 1.25 μM for SPV122. Long-term colony formation assays have been performed treating M14 melanoma cells two times a week with 250 nM of a BRAFi and at every time point cells have been fixed using crystal violet staining as previously done [10]. For clonogenic assays, Zidovudine and Stavudine NRTIs and SPV122 and Efavirenz NNRTIs have been tested starting from 100 μM and then diluted 1:2 for 10 times. Encorafenib as BRAFi, MEK162 as MEKi, Efavirenz, Stavudine and Zidovudine have been obtained by Selleckchem. SPV122 was prepared as described [29].

### Cell proliferation and in vitro colony formation assays

Viability of cells was examined with 3-(4,5-dimethylthiazol-2-yl) − 2,5-diphenyltetrazolium bromide Cell Titer 96 AQueous One Solution Cell Proliferation Assay (Promega), according to the manufacturer’s protocol. Colony formation assays have been performed by crystal violet staining as previously described [10].

### FACS analyses

Annexin V assay for apoptosis, cell cycle and mitochondrial membrane depolarization analyses have been performed in melanoma cells treated with the aforementioned inhibitors for 48 h as described in our previous work [9] using a specific Millipore kit according to the manufacturer’s protocol.

### Antibodies and Western blot

PARP and pH2A.X were purchased from Cell Signaling Technology (Boston, MA, USA). p27 and GAPDH were obtained from Santa Cruz Biotechnology (Dallas, TX, USA). Anti-rabbit and anti-mouse were purchased from AbCam (Cambridge, UK). Melanoma cells were lysed with RIPA buffer purchased by Sigma-Aldrich (St. Louis, MO, USA) and total proteins were run using Invitrogen Bolt Bis-Tris 4–12% Plus gels precast polyacrylamide gels as previously described [12].

### Immunofluorescence analyses

For immunofluorescence analyses cells were fixed with 4% paraformaldehyde (PFA; Sigma-Aldrich), permeabilized in 0.1% Triton-X100 (Sigma-Aldrich, Milan, Italy), after washing two times with PBS the cells were stained with pH2A.X (1:100 dilution) or PBS alone as negative control and incubated at 4 °C overnight. Next day, cells were washed by PBS three times to remove unbound antibodies, then secondary antibody (1:300 dilution) was added in the dark and incubated at room temperature for 1 h. Then cells were stained with Hoechst 33,342 (1:1000 dilution) for 5 min in the dark. Immunofluorescence images of cell lines were performed as previously described [6, 31]. For the pH2A.X foci counts at least 8–10 fields were randomly captured from each experimental triplicate [32].

### Statistical analyses

All results shown are presented as mean values from three independent experiments. Quantitative analysis for curve fitting and *p*-value estimation (significance *p* < 0.05) were performed by GraphPad Prism 7 (San Diego, CA, USA) [33].

## Results and discussion

### SPV122 + MAPKi reduce cell proliferation, induce apoptosis, and cell cycle blockade and delay drug resistance in BRAF-mutant melanoma cells in vitro

SPV122 and its stereoisomers have demonstrated the capability to inhibit cell proliferation and to induce differentiation in A379 melanoma cells. Those effects reproduced those observed with other NNRTIs, like efavirenz or after RNA interference (RNAi)-mediated silencing of the RT-encoding LINE-1 elements [27–29]. This has been the starting point for the present study. Clonogenic assays (shown as Additional file 1: Figure S1) performed on M14 as a representative BRAF-mutant melanoma cell line confirmed these findings. In detail, cells were treated with efavirenz and SPV122 (as NNRTIs) or stavudine and zidovudine (as NRTIs) starting from 100 μM and then diluted 1:2 for 10 times. First of all, NNRTIs demonstrated to be more effective in inhibiting M14 cell proliferation as compared to NRTIs, which are able to affect cell viability only at very high concentrations (Additional file 1: Figure S1). Furthermore, as previously reported [29] SPV122 demonstrated to be more powerful in inhibiting melanoma cell growth as compared to efavirenz (Additional file 1: Figure S1 upper plates).

**Fig. 1:**
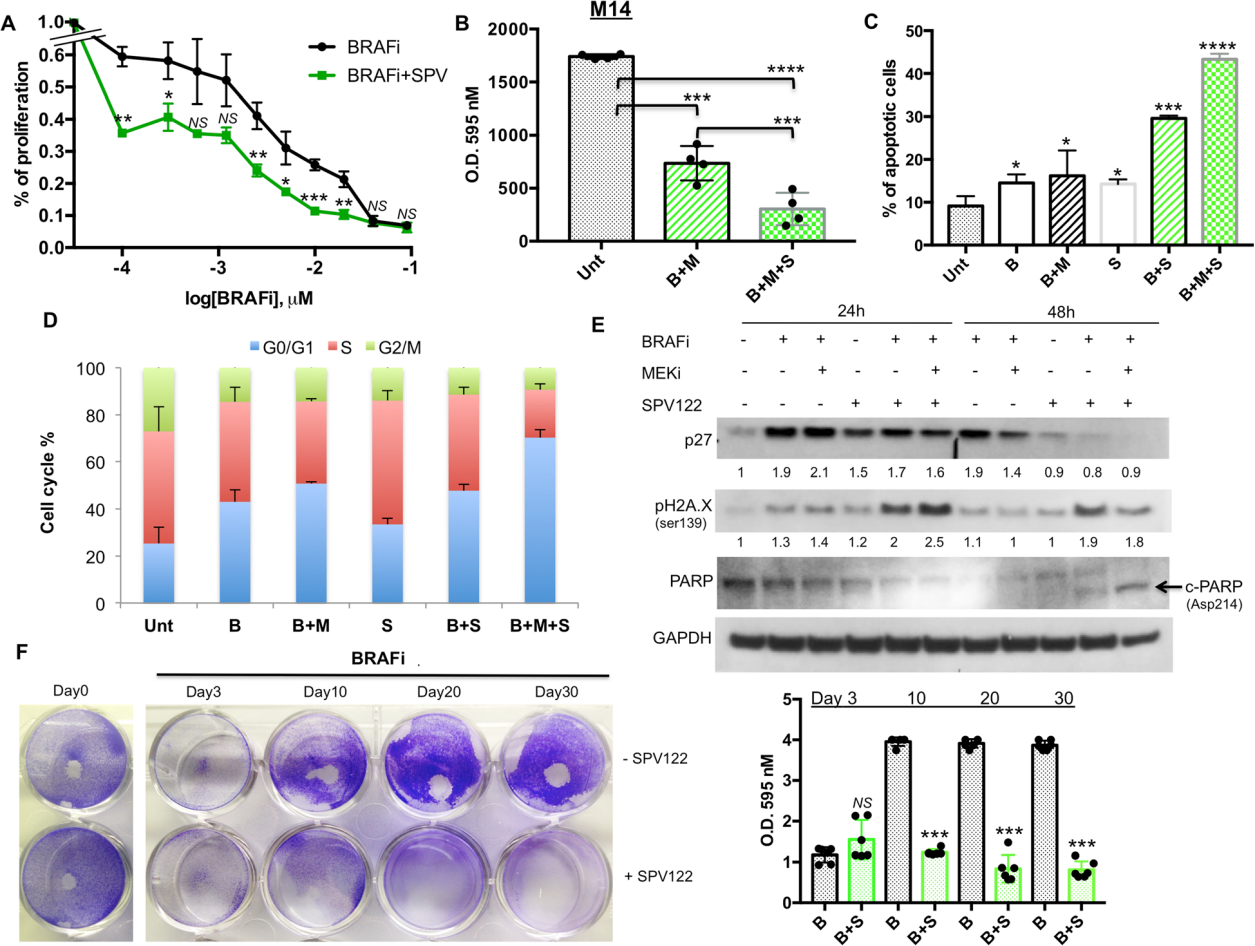
SPV122 potentiates MAPKi effects on M14 BRAF-mutant melanoma cells. **a** M14 melanoma cells have been exposed to encorafenib (BRAFi) starting from 5 μM and then diluted 1:2 for 10 times in the presence or not of SPV122 at fixed dose of 1.25 μM to measure cell viability through MTT assay after 72 h. **b** Crystal violet staining and O.D. at 595 nM reading by spectrometer assessed the growth inhibitory effects of encorafenib (BRAFi, 150 nM) and MEK162 (MEKi, 75 nM) in the presence or not of SPV122 (1.25 μM) for 72 h. The same drugs alone or in combination have been tested for apoptosis induction (**c**) and cell cycle (**d**) after 48 h of exposure. **e** M14 cells have been treated with the different drugs as previously described and total protein extracts have been subjected to Western Blot analysis to measure the expression levels of the indicated molecular effectors. **f** The same cells have been exposed two times a week with 250 nM of a BRAFi and then stained with Crystal violet (day 0). The remaining plates were treated with encorafenib in the presence or not of SPV122 (1.25 μM) and then stained after 3, 10, 20 and 30 days (left part). Quantification of data has been obtained as described above (right part)

We next started to assess the effect of combinatorial treatments of MAPKi+SPV122 using different biological assays in M14 cells. Towards this goal we tested different concentrations of encorafenib (as a BRAFi) in the presence or not of SPV122. Our results clearly indicate that this NNRTI was able to potentiate BRAFi activity on melanoma cell growth (Fig. 1a). Of note the same results were obtained in two additional BRAF-mutant melanoma cell lines, namely A375 and WM115 (Additional file 2: Figure S2A). Furthermore, as further control of the magnitude of our findings we also tested the effects of the aforementioned additional NNRTI, i.e. efavirenz in combination with a BRAFi on M14 cells. This compound has largely demonstrated to be able to exert antitumor effects on melanoma cells [34, 35] although never in the presence of BRAF inhibitors. Results confirmed the capability of this class of molecule to potentiate BRAFi activity on M14 cells (Additional file 2: Figure S2B). It is important to point out that SPV122 alone, as reported by Sbardella et al. [29], is able to trigger an efficient inhibition of melanoma cell growth only at doses higher than 1 μM (see Additional file 2: Figure S2C). Hereafter, we also tested the triple combination of BRAFi + MEKi + SPV122 on M14 cell proliferation. Pleasingly, we observed a strong improvement of the growth inhibitory in the presence of the NNRTI (Fig. 1b). The same findings were confirmed on apoptosis induction measured by FACS analyses through annexin V assays (Fig. 1c). Furthermore, in line with previous findings indicating the capability of MAPKi [9] and NNRTIs to induce a G0/G1 arrest in cancer cells [23] we observed that the triple combination induces a dramatic block in this phase in melanoma cells as compared to double and single treatments (Fig. 1d). Hereafter, we decided to investigate through Western Blot analyses the molecular effectors involved in this cell cycle blockade. Our results confirmed that MAPKi was able to induce p27 cell cycle controller [9] after 24 h of treatment despite the addition of SPV122 did not further increase this protein (Fig. 1e). Of note, after 48 h we observed a reduction of p27 both in double (BRAFi+SPV) and triple combinations (BRAFi+MEKi+SPV) probably due to the activation of apoptosis (see Fig. 1c). These results suggest the involvement of different mechanism of cell cycle arrest. For this reason, we decided to measure phosphorylated-H2AX, a typical marker of DNA double strand breaks [36] which is known to be induced by NNRTIs [23, 30]. Coherently, our results demonstrated a strong increase of this marker in SPV122 combination with MAPKi both at 24 and 48 h of treatments (Fig. 1e). Furthermore, at this last time point we also observed the cleavage of PARP protein (Fig. 1e). Whole blots are available as Additional file 3: Figure S3. All together these events may explain the strong level of apoptosis induction observed in Fig. 1c. It is important to point out that similar results on apoptosis and DNA damage induction have been previously observed on melanoma cells using another NRTI, namely azidothymidine (AZT) [26].

Finally, we sought to determine the potential impact of SPV122 on BRAFi acquired resistance in vitro. Hence, using long term in vitro colony formation assays, we tested the consequence of SPV122 on M14 BRAFi-sensitive melanoma cells exposed constantly to encorafenib for 28 d. Our data demonstrate that melanoma cells co-treated with SPV122 completely loose the ability to form BRAFi-resistant colonies (Fig. 1f). In contrast, in the absence of this inhibitor cells were initially affected by exposure to encorafenib (day 3 of drug exposure), but very rapidly they succeeded in the establishment of resistant colonies (Fig. 1f). These findings suggest the possibility that NNRTIs may potentiate the efficacy of current targeted therapies for BRAF-mutant melanomas patients and may delay the establishment of drug resistance.

### SPV122 + MAPKi induce DNA damage coupled with mitochondrial membrane depolarization and ROS production in BRAF-mutant melanoma cells

**Fig. 2:**
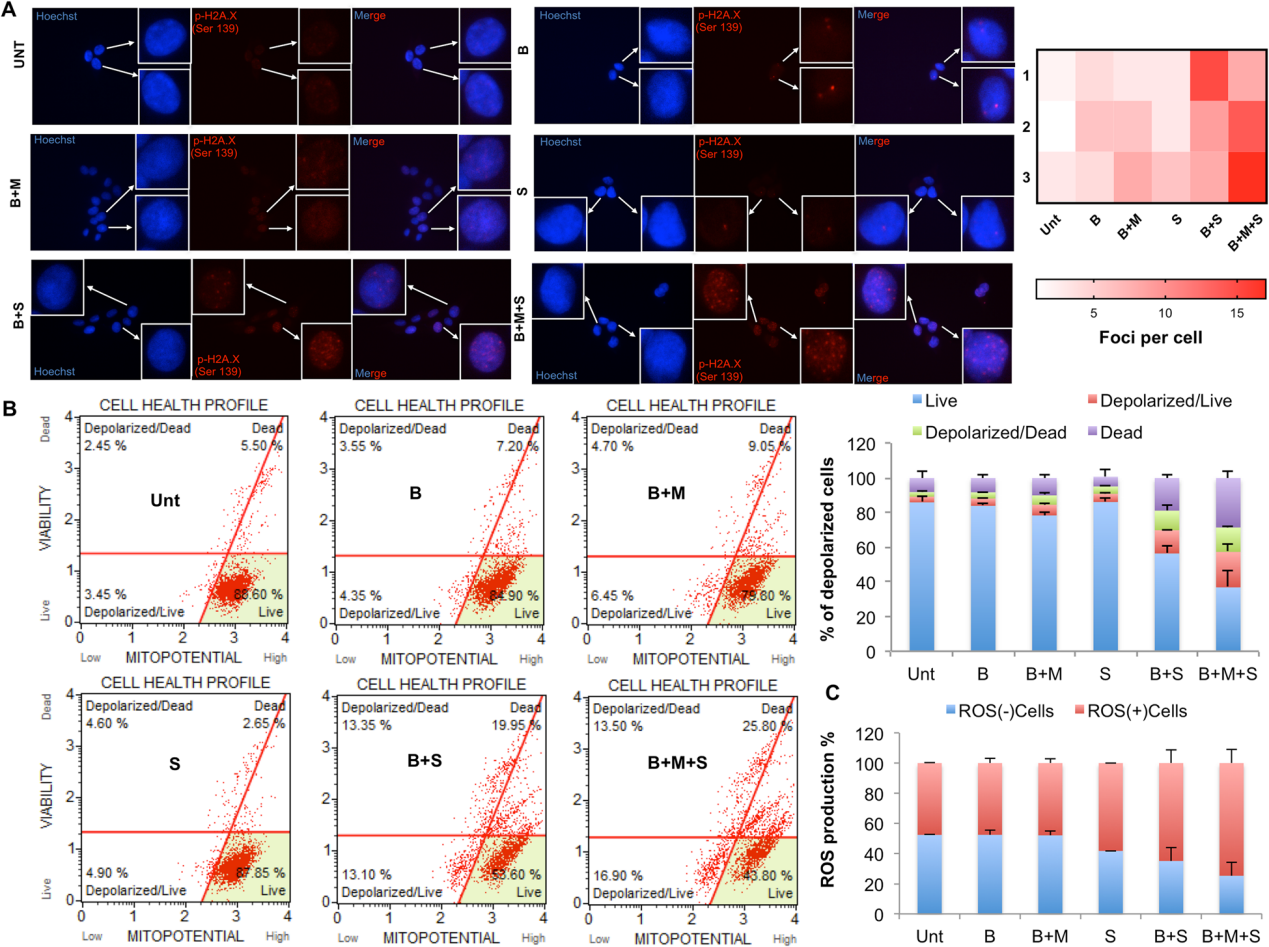
SPV122 + MAPKi induce DNA damage, mitochondrial membrane depolarization and ROS production in M14 cells. **a** Immunofluorescence analyses have been performed to quantify nuclear p-H2A.X as a marker of DSBs upon exposure to the aforementioned combinatorial regimens for 24 h. Scale bars: 50 μm; 40x magnification. Heat map has been plotted to quantify the number of foci per cell. **b** Mitochondrial membrane depolarization has been assessed by FACS analyses after 48 h of exposure of M14 cells to encorafenib, MEK162 and/or SPV122 (left panels). Data have been plotted to assess the % of depolarized cells (right part). **c** M14 cells treated as described above have been tested for ROS production by FACS analyses

DNA damage may mechanistically explain the strong inhibitory effects obtained when we add SPV122 to MAPKi on BRAF-mutant melanoma cells. To strengthen these findings, we sought to measure the accumulation of nuclear phosphorylated-H2A.X by immunofluorescence [36, 37]. Results (Fig. 2a, left panels) clearly show the increase of H2A.X foci corresponding to DNA double-strand breaks sites when we combine SPV122 with either BRAFi alone or to greater extent with both a BRAF and a MEK inhibitor. Data quantification of foci formation per cell was plotted in a heat-map diagram (Fig. 2a right panel). Of note the same findings were observed also in A375 cells (Additional file 4: Figure S4). Hereafter, we assess whether the depolarization of mitochondrial inner transmembrane potential as a marker of apoptosis [38, 39] may occur in different combinatorial regimens. Data indicate that this was indeed the case. FACS analyses and their quantification (Fig. 2b, left and right panels respectively) demonstrated dramatic effects on the alteration of the mitochondrial inner membrane potential in the combination of SPV122 with MAPKi. Again, the most impressive results were obtained in the case of triple combinations of BRAFi + MEKi + SPV122 (Fig. 2b). The same data were obtained also in A375 cells (Additional file 5: Figure S5). Of note, similar results on mitochondrial dysfunction have been previously observed by treating cancer cells with the aforementioned NRTI, i.e. AZT [40, 41].

Finally, we reasoned that the thread linking between the DNA damage, the alteration of mitochondrial membrane potential and induction of apoptosis may be the accumulation of oxidative stress in melanoma cells [42, 43]. Towards this goal, we took advantage of FACS analyses using dihydroethidium (DHE), a reagent that measures the reactive oxidative species allowing to distinguish cellular subpopulations into ROS positive (+) or negative (−) cells. Results clearly show that SPV122 addition to BRAFi and even more to BRAFi + MEKi dramatically increased the number of ROS (+) cells (Fig. 2c). All together these findings demonstrate that the powerful growth inhibitory effects obtained by the combination of NNRTIs with target therapy in melanoma are linked to dysregulation of the mitochondrial membrane potential and ROS production which in turn results in excessive DNA damage and apoptosis.

## Conclusions

Reverse Transcriptase Inhibitors have demonstrated efficacy as anticancer agents; however, their role as potentiators of target therapies have not been addressed yet. Here, using as working models BRAF-mutant melanoma cells we have demonstrated for the first time that the RT inhibitor SPV122 strongly synergizes with MAPKi to reduce cell proliferation, induce apoptosis and delay drug resistance in vitro. Of course, these results warrant further corroboration in in vivo tumor models. Furthermore, the molecular mechanisms underpinning NNRTIs’ efficacy in cancer cells still needs to be further elucidated. In this context, very recently Bellisai et al. [30] have reported that SPV122 decreased proliferation of metastatic prostate carcinoma cells by concomitant induction of genomic DNA damage. In line with these findings, we observed that SPV122 addition to MAPKi potently provokes DSBs as highlighted by nuclear accumulation of H2A.X. Mechanistically, we provide evidences that these events may be caused by the alteration of the inner transmembrane mitochondrial potential coupled with ROS production.

We are aware that our data do not directly allow to correlate SPV122 effects as potentiator of BRAF and MEK inhibitors in melanoma, with inhibition of retrotransposon activity. In this regard we can envisage the involvement of three possible elements. 1) Human endogenous retrovirus type K of HML-2 family (HK2) members which are known to be transcriptionally active [44]. Some virus-like particles of this family have been detected in breast cancer, leukemia and also in melanoma [45]. However, K103 enzyme, which is the only HK2 functional RT has been demonstrated to be inhibited only by NRTIs and not by NNRTIs in melanoma cell lines in vitro [45]. These evidences tend to exclude the involvement of HERVs in our working model. 2) Telomerase activation, which is a hallmark of advanced malignancies including 67–85% of metastatic melanomas [46]. Interestingly, it has been recently demonstrated that oncogenic BRAF-V600-MAPK signaling potently activates a mutant TERT promoter, which in turn up-regulates TERT expression [47, 48]. This event encompasses not only the classical telomerase function but also a strong suppression of MAPKi-induced melanoma cell apoptosis [47, 48]. In the light of these evidences TERT aberrant expression may impact on the establishment of resistance to target therapy in BRAF-mutant melanomas. However, also in this case NNRTIs such as our SPV122 have not demonstrated the ability to inhibit telomerase activity, even at millimolar concentrations [49]. Furthermore, only long-term and continuous treatments with NRTIs resulted in an accelerated loss of telomere repeats and antiproliferative effects in colorectal cancer and melanoma cell lines in vitro [26]. 3) LINE-1 insertions, which have been observed and mapped in the genomes of several human cancers [50]. Furthermore, their activation has been reported to orchestrate melanoma cell transcriptome by sequestering RNAs through the reverse transcriptase activity to give rise to aberrant RNA:DNA hybrids [12]. Hence it is not surprisingly that the inhibition of LINE-1 expression through RNA interference (RNAi) is able to reduce proliferation and to restore differentiation of melanoma and prostate cancer cells [27–30]. These effects have been reproduced treating the same cells with different NNRTIs in vitro, such as nevirapine, efavirenz as well as SPV122 and its stereoisomers [27–29]. This suggests that the anti-tumoral activities of these compounds may not reflect a random off target consequence. Finally, the effects of the different aforementioned NNRTIs are induced quite rapidly (within a few days), supporting the conclusion that all tested molecules share a common target in melanoma cells.

In conclusion, it is evident that additional efforts to directly correlate the RT inhibitor SPV122 with retrotransposons activity in melanoma cells are needed. However, the strength of our work encompasses the demonstration of the capability of RTIs to delay resistance to target therapy in BRAF-mutant melanoma cells in vitro. These findings pave the way for further testing of SPV122 combinations with MAPKi in in vivo mouse xenograft models, where it will be possible to assess the capability of these new triple combinations to control tumor recurrence for longer time.

## Supplementary information

**Additional file 1 : Figure S1.** M14 cells treated with different RTIs. Cells have been exposed for 72 h to perform clonogenic assays in the presence of SPV122 or Efavirenz (as NNRTIs, upper plates) and Zidovudine or Stavudine (as NRTIs, lower plates). Inhibitors have been used starting from 100 μM and then diluted 1:2 for ten times.

**Additional file 2 : Figure S2.** Effects of RTIs alone or in combination with BRAFi in different BRAF-mutant melanoma cells. (**A**) A375 and WM115 have been exposed to encorafenib (BRAFi) starting from 5 μM and then diluted 1:2 for 10 times in the presence or not of SPV122 at fixed dose of 1.25 μM to measure cell viability through MTT assay after 72 h. (**B)** The same experimental approaches have been performed in M14 cells in the presence of a BRAFi and/or efavirenz used at of 2.5 μM. (**C)** M14, A375 and WM115 cells have been treated with SPV122 starting from 5 μM and then diluted 1:2 for 10 times to measure cell viability through MTT assay after 72 h.

**Additional file 3 : Figure S3**. Whole blots of Fig. 1e.

**Additional file 4 : Figure S4**. SPV122 + MAPKi induce DNA damage in A375 cells. Immunofluorescence analyses have been performed to quantify nuclear p-H2A.X upon exposure to the aforementioned combinatorial regimens for 24 h. Scale bars: 50 μm; 40x magnification.

**Additional file 5 : Figure S5**. SPV122 + MAPKi induce mitochondrial membrane depolarization in A375 cells. Mitochondrial membrane depolarization has been assessed by FACS analyses after 48 h of exposure to encorafenib, MEK162 and/or SPV122.

## Data Availability

All data used in this study are available from the corresponding author on reasonable requests.

## Abbreviations

MAPKi: BRAF/MEK inhibitors
LINE-1: Long Interspersed Nuclear Element1
RTi: Reverse transcriptase inhibitors
NRTIs: Nucleoside RT inhibitors
NNRTIs: Non-nucleoside RT inhibitors
FBS: Fetal Bovine Serum

## References

[1] Domingues B, Lopes JM, Soares P, Pópulo H (2018). Melanoma treatment in review. Immunotargets Ther.

[2] Dummer R, Ascierto PA, Gogas HJ, Arance A, Mandala M, Liszkay G (2018). Encorafenib plus binimetinib versus vemurafenib or encorafenib in patients with BRAF-mutant melanoma (COLUMBUS): a multicentre, open-label, randomised phase 3 trial. Lancet Oncol.

[3] Fattore L, Sacconi A, Mancini R, Ciliberto G (2017). MicroRNA-driven deregulation of cytokine expression helps development of drug resistance in metastatic melanoma. Cytokine Growth Factor Rev.

[4] Hong A, Moriceau G, Sun L, Lomeli S, Piva M, Damoiseaux R (2018). Exploiting drug addiction mechanisms to select against MAPKi-resistant melanoma. Cancer Discov.

[5] Hugo W, Shi H, Sun L, Piva M, Song C, Kong X (2015). Non-genomic and immune evolution of melanoma acquiring MAPKi resistance. Cell.

[6] Pisanu ME, Maugeri-SaccÃ M, Fattore L, Bruschini S, De Vitis C, Tabbì E (2018). Inhibition of Stearoyl-CoA desaturase 1 reverts BRAF and MEK inhibition-induced selection of cancer stem cells in BRAF-mutated melanoma. J Exp Clin Cancer Res.

[7] Fattore L, Ruggiero CF, Liguoro D, Mancini R, Ciliberto G (2019). Single cell analysis to dissect molecular heterogeneity and disease evolution in metastatic melanoma. Cell Death Dis.

[8] Ruggiero CF, Malpicci D, Fattore L, Madonna G, Vanella V, Mallardo D (2019). ErbB3 phosphorylation as central event in adaptive resistance to targeted therapy in metastatic melanoma: early detection in CTCs during therapy and insights into regulation by Autocrine Neuregulin. Cancers (Basel).

[9] Fattore L, Mancini R, Acunzo M, Romano G, LaganÃ A, Pisanu ME (2016). miR-579-3p controls melanoma progression and resistance to target therapy. Proc Natl Acad Sci U S A.

[10] Fattore L, Ruggiero CF, Pisanu ME, Liguoro D, Cerri A, Costantini S (2019). Reprogramming miRNAs global expression orchestrates development of drug resistance in BRAF mutated melanoma. Cell Death Diff.

[11] Fattore L, Campani V, Ruggiero CF, Salvati V, Liguoro D, Scotti L (2020). In vitro biophysical and biological characterization of lipid nanoparticles co-encapsulating Oncosuppressors miR-199b-5p and miR-204-5p as Potentiators of target therapy in metastatic melanoma. Int J Mol Sci.

[12] Sciamanna I, De Luca C, Spadafora C (2016). The reverse transcriptase encoded by LINE-1 Retrotransposons in the genesis, progression, and therapy of Cancer. Front Chem.

[13] Bastos M, Costa CCP, Bezerra TC, da Silva FC, Boechat N (2016). Efavirenz a nonnucleoside reverse transcriptase inhibitor of first-generation: Approaches based on its medicinal chemistry. Eur J Med Chem.

[14] Goodier JL (2016). Restricting Retrotransposons: A review. Mob DNA.

[15] Attermann AS, Bjerregaard A-M, Saini SK, Grønbæk K, Hadrup SR (2018). Human endogenous retroviruses and their implication for Immunotherapeutics of Cancer. Ann Oncol.

[16] Grandi N, Tramontano E (2018). Human endogenous retroviruses are ancient acquired elements still shaping innate immune responses. Front Immunol.

[17] Rodić N, Burns KH (2013). Long interspersed element-1 (LINE-1): passenger or driver in human neoplasms?. PLoS Genet.

[18] Shukla R, Upton KR, Muñoz-Lopez M, Gerhardt DJ, Fisher ME, Nguyen T (2013). Endogenous retrotransposition activates oncogenic pathways in hepatocellular carcinoma. Cell.

[19] Cajuso T, Sulo P, Tanskanen T, Katainen R, Taira A, Hänninen UA (2019). Retrotransposon insertions can initiate colorectal cancer and are associated with poor survival. Nat Commun.

[20] Scott EC, Gardner EJ, Masood A, Chuang NT, Vertino PM, Devine SE (2016). A hot L1 retrotransposon evades somatic repression and initiates human colorectal cancer. Genome Res.

[21] Cheng D, Zhao Y, Zhang F, Zhang J, Wang S, Zhu J (2019). Engineering a humanized telomerase reverse transcriptase gene in mouse embryonic stem cells. Sci Rep.

[22] Jafri MA, Ansari SA, Alqahtani MH, Shay JW (2016). Roles of telomeres and telomerase in Cancer, and advances in telomerase-targeted therapies. Genome Med.

[23] Hecht M, Harrer T, Körber V, Sarpong EO, Moser F, Fiebig N (2018). Cytotoxic effect of Efavirenz in BxPC-3 pancreatic cancer cells is based on oxidative stress and is synergistic with ionizing radiation. Oncol Lett.

[24] Brüning A, Jückstock J, Kost B, Tsikouras P, Weissenbacher T, Mahner S (2017). Induction of DNA damage and apoptosis in human leukemia cells by efavirenz. Oncol Rep.

[25] Carlini F, Ridolfi B, Molinari A, Parisi C, Bozzuto G, Toccacieli L (2010). The reverse transcription inhibitor abacavir shows anticancer activity in prostate cancer cell lines. PLoS One.

[26] Aschacher T, Sampl S, Käser L, Bernhard D, Spittler A, Holzmann K (2012). The combined use of known antiviral reverse transcriptase inhibitors AZT and DDI induce anticancer effects at low concentrations. Neoplasia.

[27] Bartolini S, Mai A, Artico M, Paesano N, Rotili D, Spadafora C (2005). 6-[1-(2,6-Difluorophenyl)ethyl] pyrimidinones antagonize cell proliferation and induce cell differentiation by inhibiting (A Nontelomeric) endogenous reverse transcriptase. J Med Chem.

[28] Sbardella G, Bartolini S, Castellano S, Artico M, Paesano N, Rotili D (2006). 6-alkylthio-4-[1-(2,6-difluorophenyl)alkyl]-1H-[1,3,5]triazin-2-ones (ADATs): novel regulators of cell differentiation and proliferation. ChemMedChem.

[29] Sbardella G, Mai A, Bartolini S, Castellano S, Cirilli R, Rotili D (2011). Modulation of cell differentiation, proliferation, and tumor growth by dihydrobenzyloxopyrimidine non-nucleoside reverse transcriptase inhibitors. J Med Chem.

[30] Bellisai C, Sciamanna I, Rovella P, Giovannini D, Baranzini M, Pugliese GM (2020). Reverse transcriptase inhibitors promote the remodelling of nuclear architecture and induce autophagy in prostate cancer cells. Cancer Lett.

[31] Bruschini S, di Martino S, Pisanu ME, Fattore L, De Vitis C, Laquintana V (2020). CytoMatrix for a reliable and simple characterization of lung cancer stem cells from malignant pleural effusions. J Cell Physiol.

[32] Burdak-Rothkamm S, Short SC, Folkard M, Rothkamm K, Prise KM (2007). ATR-dependent radiation-induced gamma H2AX foci in bystander primary human astrocytes and glioma cells. Oncogene.

[33] De Vitis C, Corleone G, Salvati V, Ascenzi F, Pallocca M, De Nicola F (2019). B4GALT1 is a new candidate to maintain the Stemness of lung Cancer stem cells. J Clin Med.

[34] Sciamanna I, Landriscina M, Pittoggi C, Quirino M, Mearelli C, Beraldi R (2005). Inhibition of endogenous reverse transcriptase antagonizes human tumor growth. Oncogene..

[35] Lugini L, Sciamanna I, Federici C, Iessi E, Spugnini EP, Fais S (2017). Antitumor effect of combination of the inhibitors of two new oncotargets: proton pumps and reverse transcriptase. Oncotarget.

[36] Salguero I, Belotserkovskaya R, Coates J, Sczaniecka-Clift M, Demir M, Jhujh S (2019). MDC1 PST-repeat region promotes histone H2AX-independent chromatin association and DNA damage tolerance. Nat Commun.

[37] Lustri AM, Di Matteo S, Fraveto A, Costantini D, Cantafora A, Napoletano C (2017). TGF-Î^2^ signaling is an effective target to impair survival and induce apoptosis of human cholangiocarcinoma cells: A study on human primary cell cultures. PLoS One.

[38] Wang C, Youle RJ (2009). The role of mitochondria in apoptosis*. Annu Rev Genet.

[39] Dijk SN, Protasoni M, Elpidorou M, Kroon AM, Taanman J (2020). Mitochondria as target to inhibit proliferation and induce apoptosis of cancer cells: the effects of doxycycline and gemcitabine. Scientific Rep.

[40] Mattson DM, Ahmad IM, Dayal D, Parsons AD, Aykin-Burns N, Li L (2009). Cisplatin combined with Zidovudine enhances cytotoxicity and oxidative stress in human head and neck Cancer cells via a Thiol-dependent mechanism. Free Radic Biol Med.

[41] Kline ER, Bassit L, Hernandez-Santiago BI, Detorio MA, Liang B, Kleinhenz DJ (2009). Long-term exposure to AZT, but not d4T, increases endothelial cell oxidative stress and mitochondrial dysfunction. Cardiovasc Toxicol.

[42] Bayurova E, Jansons J, Skrastina D, Smirnova O, Mezale D, Kostyusheva A (2019). HIV-1 reverse transcriptase promotes tumor growth and metastasis formation via ROS-dependent upregulation of twist. Oxid Med Cell Longev.

[43] Kim SJ, Kim HS, Seo YR (2019). Understanding of ROS-inducing strategy in anticancer therapy. Oxid Med Cell Longev.

[44] Ma W, Hong Z, Liu H, Chen X, Ding L, Liu Z (2016). Human endogenous retroviruses-K (HML-2) expression is correlated with prognosis and Progress of hepatocellular carcinoma. Biomed Res Int.

[45] Contreras-Galindo R, Dube D, Fujinaga K, Kaplan MH, Markovitz DM (2017). Susceptibility of human endogenous retrovirus type K to reverse transcriptase inhibitors. J Virol.

[46] Shay JW (2016). Role of telomeres and telomerase in aging and Cancer. Cancer Discov.

[47] Liu R, Zhang T, Zhu G, Xing M (2018). Regulation of mutant TERT by BRAF V600E/MAP kinase pathway through FOS/GABP in human Cancer. Nat Commun.

[48] Tan J, Liu R, Zhu G, Umbricht CB, Xing M (2020). TERT promoter mutation determines apoptotic and therapeutic responses of BRAF-mutant cancers to BRAF and MEK inhibitors: Achilles heel. Proc Natl Acad Sci U S A.

[49] Hukezalie KR, Thumati NR, Côté HCF, Wong JMY (2012). In vitro and ex vivo inhibition of human telomerase by anti-HIV nucleoside reverse transcriptase inhibitors (NRTIs) but not by non-NRTIs. PLoS One.

[50] Burns KH (2017). Transposable elements in cancer. Nat Rev Cancer.

