# Supplementary figures and images for: Reverse transcriptase inhibition potentiates target therapy in BRAF-mutant melanomas: effects on cell proliferation, apoptosis, DNA-damage, ROS induction and mitochondrial membrane depolarization
Source: Cell Commun Signal. 2020 Sep 15;18:150. doi: 10.1186/s12964-020-00633-7 (PMC7493390; doi:10.1186/s12964-020-00633-7)

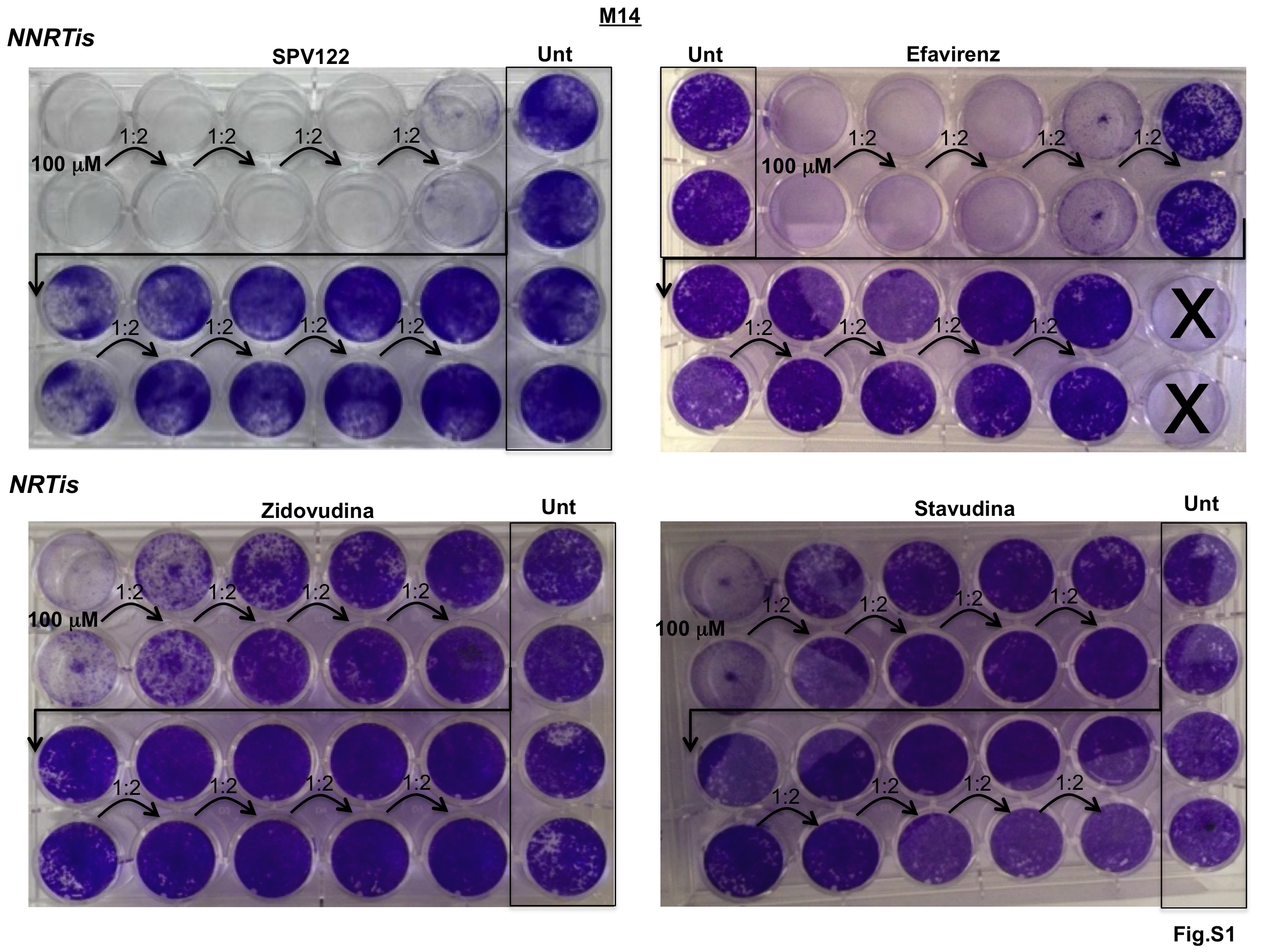

Supplement: Supplementary file 2 — Additional file 1 : Figure S1. M14 cells treated with different RTIs. Cells have been exposed for 72 h to perform clonogenic assays in the presence of SPV122 or Efavirenz (as NNRTIs, upper plates) and Zidovudine or Stavudine (as NRTIs, lower plates). Inhibitors have been used starting from 100 μM and then diluted 1:2 for ten times. [file 12964_2020_633_MOESM2_ESM.tiff]

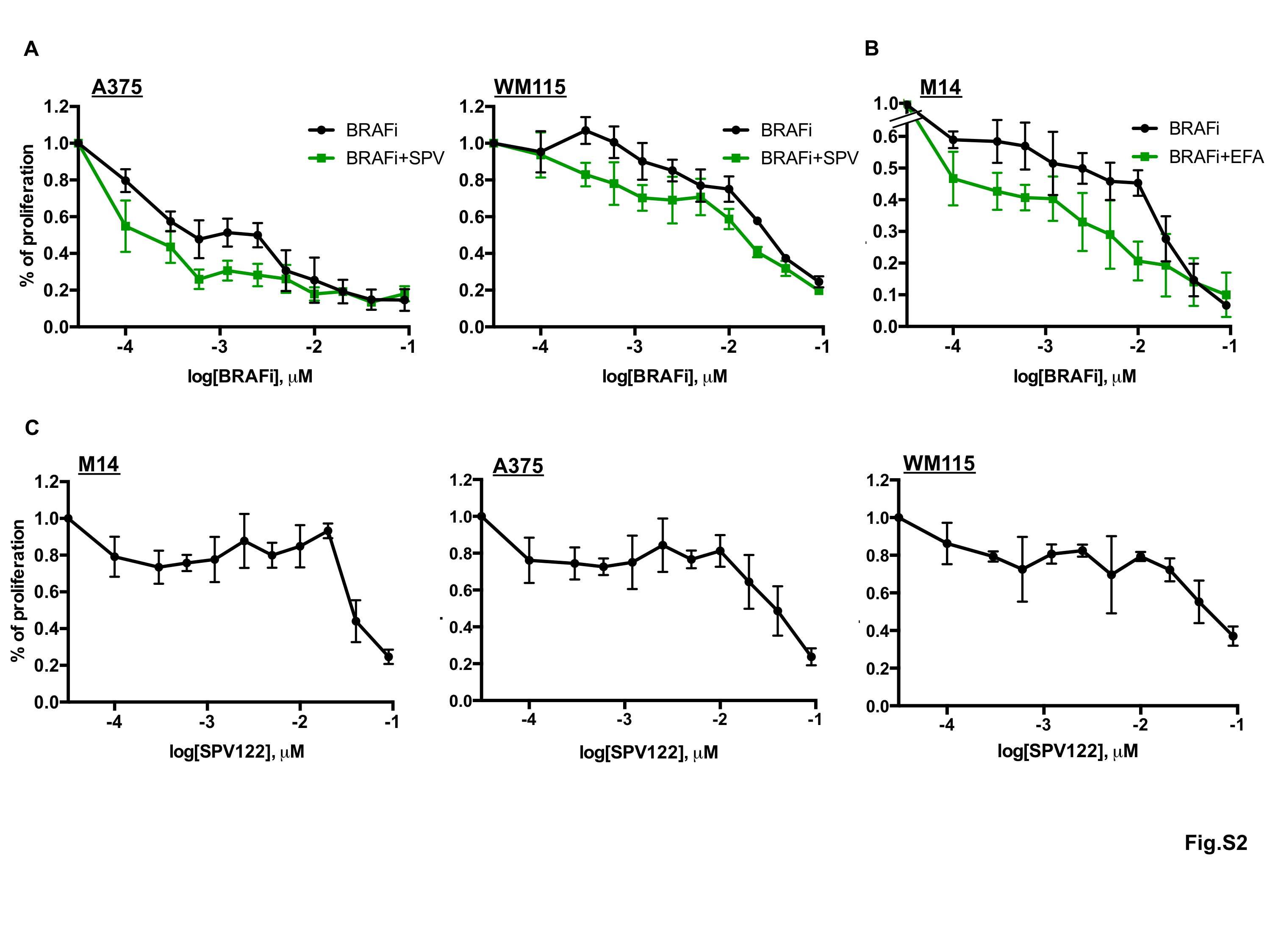

Supplement: Supplementary file 3 — Additional file 2 : Figure S2. Effects of RTIs alone or in combination with BRAFi in different BRAF-mutant melanoma cells. (A) A375 and WM115 have been exposed to encorafenib (BRAFi) starting from 5 μM and then diluted 1:2 for 10 times in the presence or not of SPV122 at fixed dose of 1.25 μM to measure cell viability through MTT assay after 72 h. (B) The same experimental approaches have been performed in M14 cells in the presence of a BRAFi and/or efavirenz used at of 2.5 μM. (C) M14, A375 and WM115 cells have been treated with SPV122 starting from 5 μM and then diluted 1:2 for 10 times to measure cell viability through MTT assay after 72 h. [file 12964_2020_633_MOESM3_ESM.tiff]

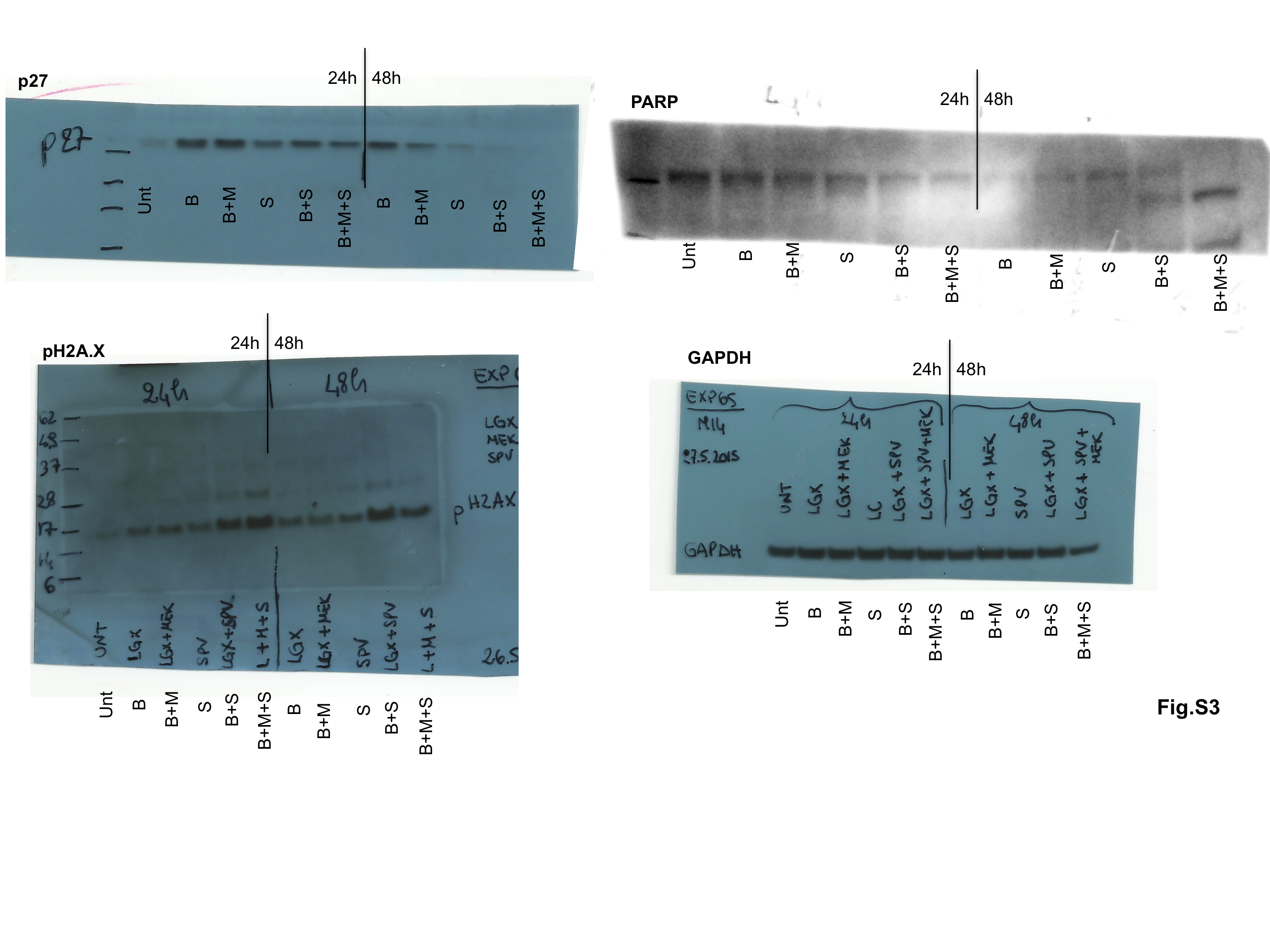

Supplement: Supplementary file 4 — Additional file 3 : Figure S3. Whole blots of Fig. 1e. [file 12964_2020_633_MOESM4_ESM.tiff]

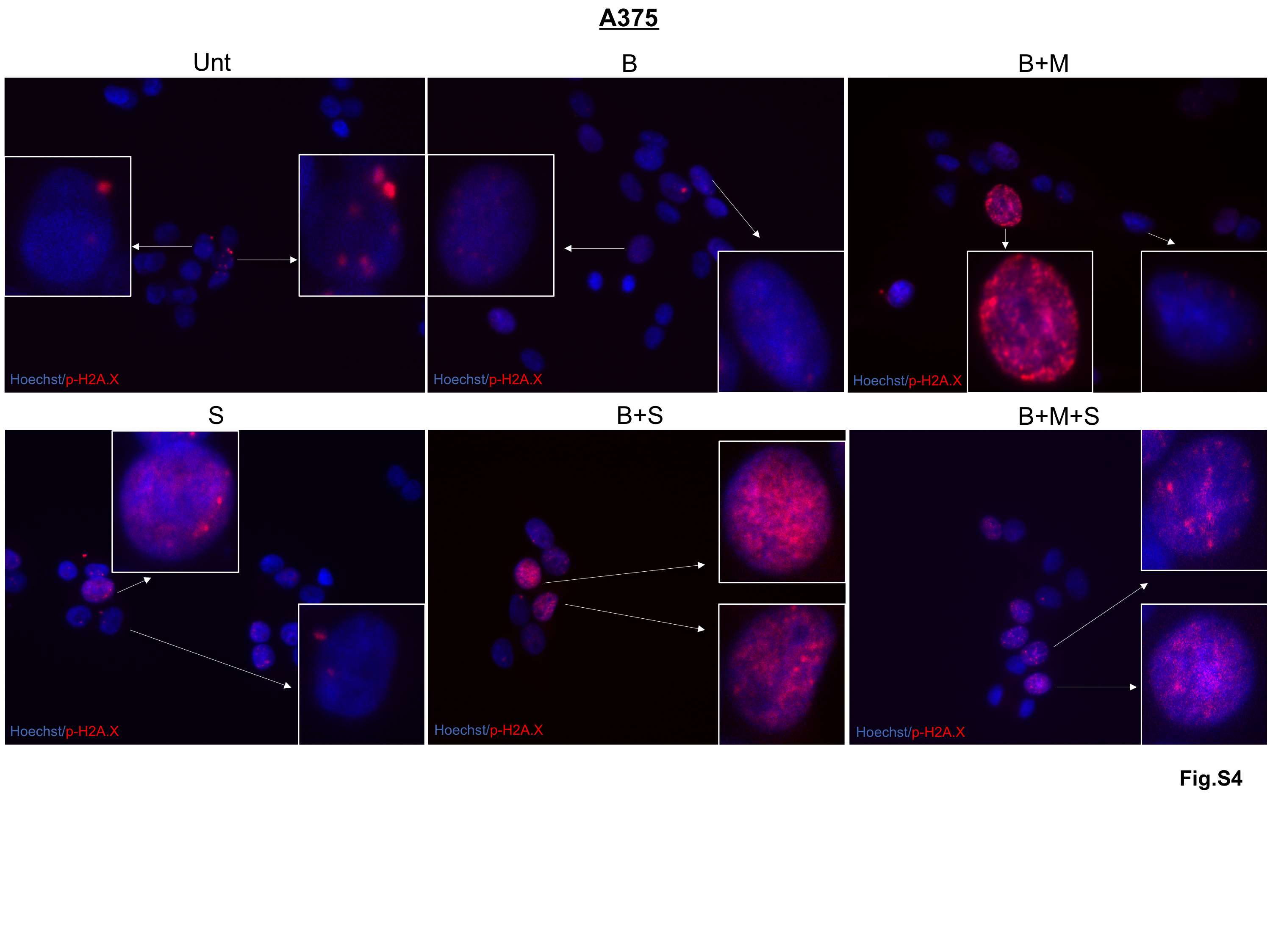

Supplement: Supplementary file 5 — Additional file 4 : Figure S4. SPV122 + MAPKi induce DNA damage in A375 cells. Immunofluorescence analyses have been performed to quantify nuclear p-H2A.X upon exposure to the aforementioned combinatorial regimens for 24 h. Scale bars: 50 μm; 40x magnification. [file 12964_2020_633_MOESM5_ESM.tiff]

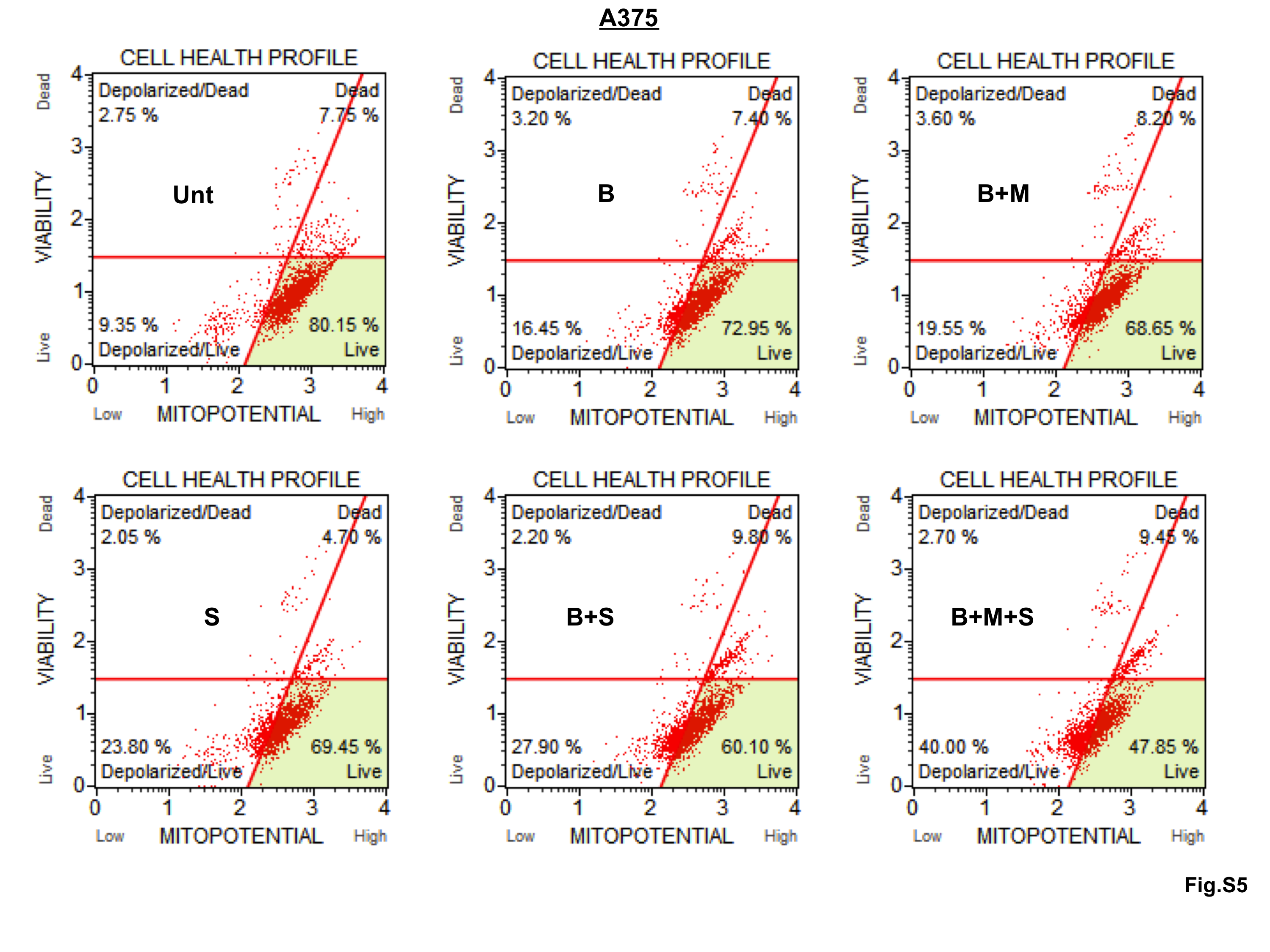

Supplement: Supplementary file 6 — Additional file 5 : Figure S5. SPV122 + MAPKi induce mitochondrial membrane depolarization in A375 cells. Mitochondrial membrane depolarization has been assessed by FACS analyses after 48 h of exposure to encorafenib, MEK162 and/or SPV122. [file 12964_2020_633_MOESM6_ESM.tiff]
